# Transaortic Alfieri stitch for mitral systolic anterior motion in acute type A dissection

**DOI:** 10.1016/j.xjtc.2026.102308

**Published:** 2026-03-07

**Authors:** Shogo Matsunaga, Masami Konnai, Takashi Igarashi, Hiroki Wakamatsu, Ken-ichi Imasaka

**Affiliations:** Department of Cardiovascular Surgery, Fukushima Medical University Hospital, Fukushima, Japan

**Figure:**
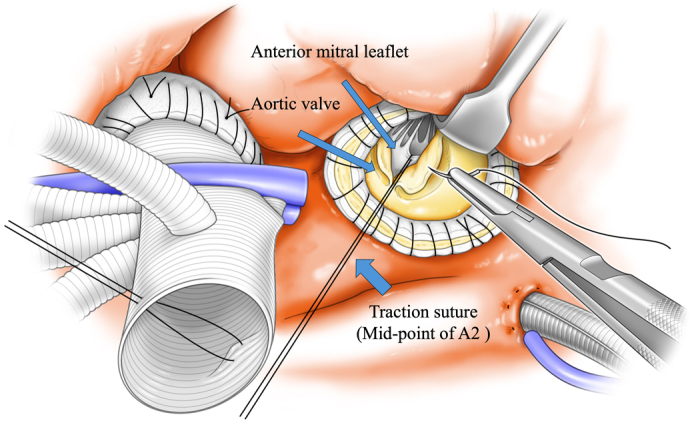
Schematic surgeon's view of the transaortic Alfieri stitch.

**Central Message**

The transaortic Alfieri stitch is a practical and reversible approach to prevent the potentially lethal combination of SAM and MR worsening in AAD surgery.

Systolic anterior motion (SAM) of the mitral valve is common in hypertrophic cardiomyopathy but is exceedingly rare in acute type A dissection (AAD). The mechanism of SAM in AAD is multifactorial, involving not only anatomical predisposition such as a sigmoid septum but also acute geometric distortion of the left ventricular outflow tract (LVOT) as a result of aortic root displacement. Because unrecognized SAM during emergent aortic repair can lead to a “catecholamine dilemma”—where inotropic support exacerbates hemodynamic instability—proactive intraoperative management is crucial. We describe a case of AAD complicated by SAM-related mitral regurgitation (MR) that was successfully treated with the transaortic Alfieri stitch.

Institutional review board approval was not required. Patient consent was obtained for publication; there is potentially identifiable information in this article.

## Case Report

**Figure 1:**
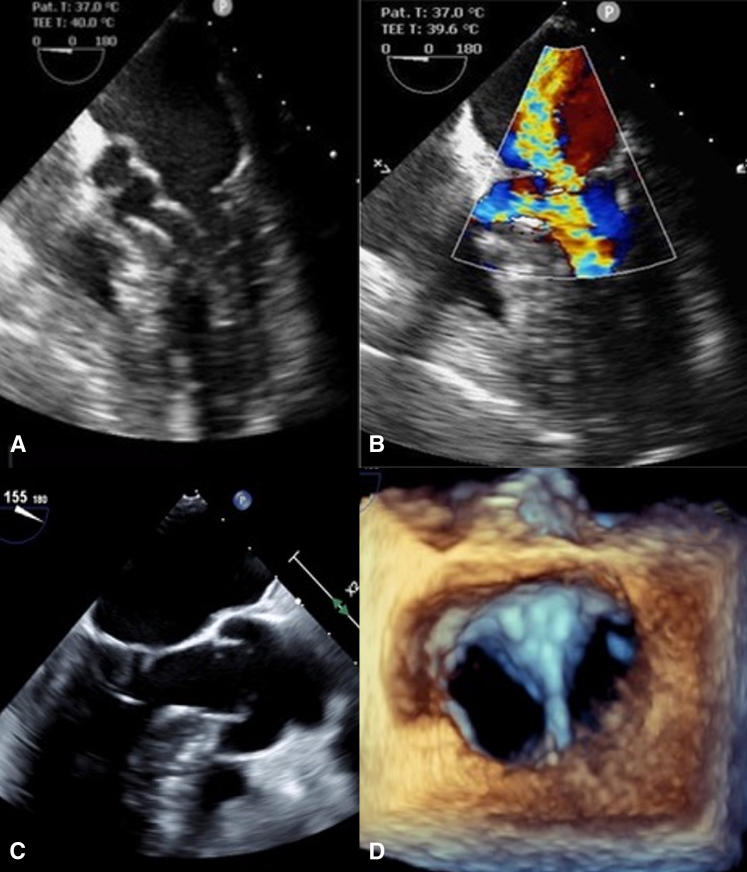
Preoperative transesophageal echocardiogram showed left ventricular outflow tract (*LVOT*) obstruction with systolic anterior motion (*SAM*) (A) and moderate mitral regurgitation (B). Postoperative imaging demonstrated resolution of LVOT obstruction and disappearance of SAM (C) after placement of an Alfieri stitch between A2 and P2 (D).

**Figure 2:**
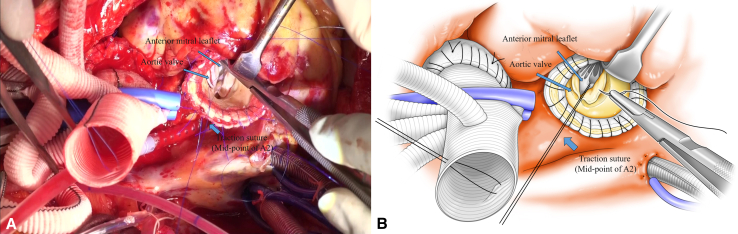
Intraoperative finding (A) and schematic of surgeon's view (B) of the transaortic Alfieri stitch.

A 67-year-old woman presented with epigastric pain attributable to AAD (Video 1). Preoperative echocardiography revealed LVOT obstruction (peak gradient 65 mm Hg) with SAM and moderate MR (Figure 1, *A* and *B*, Video 1). Although asymmetric septal hypertrophy criteria were not fully met (interventricular septum 13.6 mm, posterior wall 10 mm; ratio 1.36), a prominent sigmoid septum was noted. Crucially, the mitral valve area (MVA) was 5.0 cm^2^ and the valve was structurally normal, confirming that the MR was purely SAM-mediated. Intraoperatively, prebypass medical management (volume loading and beta-blockers) failed to alleviate the SAM. Total arch replacement was performed using the distal-first technique. After distal anastomosis with the patient under moderate hypothermic circulatory arrest with selective antegrade cerebral perfusion, we commenced lower-body perfusion. After the proximal aortic stump was reinforced, a 4-0 polypropylene stitch was placed between the midpoints of A2 and P2 through the open aortic root (Figure 2, Video 1). Subsequently, the proximal anastomosis was completed and coronary perfusion was initiated, followed by reconstruction of the arch vessels. Postoperative echocardiography showed complete resolution of SAM (Figure 1, *C* and *D*; Video 1). At 6-month follow-up, the patient remained asymptomatic with an LVOT peak pressure gradient of 3.5 mm Hg and no signs of functional mitral stenosis (mean pressure gradient: 1 mm Hg; peak velocity: 1.0 m/s).

## Discussion

SAM is exceedingly rare in AAD, and its mechanism in this setting is multifactorial. Beyond anatomical predisposition (sigmoid septum and a 30-mm anterior leaflet), acute geometric distortion of the aorta plays a crucial role. According to Rylski and colleagues,^1^ AAD significantly increases aortic tortuosity and ST-junction diameter while the sinus base remains relatively stable. This “distal expansion with proximal fixation” likely distorts the aortoventricular angle, shifting the systolic flow vector posteriorly toward the mitral leaflets. This geometric shift, synergizing with pain-induced hyperdynamic physiology, triggers SAM.

Our proactive decision to perform a transaortic Alfieri stitch was based on 2 strategic considerations. First, the “catecholamine dilemma”: we anticipated that any need for inotropic support during weaning from cardiopulmonary bypass would inevitably exacerbate LVOT obstruction. Second, the reversibility of the technique: the stitch is technically simple and can be easily divided to restore the original anatomy if intraoperative evaluation shows suboptimal.

Following our established criteria,^2^ this approach is reserved for central functional or SAM-mediated MR with preserved leaflet morphology. Organic pathologies involving commissural lesions, severe tethering, or extensive calcification remain contraindications, because these conditions require annuloplasty under optimal visualization.

To ensure safety, we suggest a preoperative MVA threshold of >4.0 cm^2^. Recent evidence indicates that a single edge-to-edge repair reduces MVA by 50% to 57%^3^; thus, a preoperative MVA >4.0 cm^2^ ensures a postoperative MVA >2.0 cm^2^, minimizing the risk of functional stenosis. This approach is highly beneficial not only in AAD but also in other surgeries requiring an aortotomy, such as aortic valve replacement, ascending aortic, or total arch replacement and septal myectomy. The transaortic Alfieri stitch offers a rapid, effective, and reversible method to stabilize hemodynamic in complex aortic emergencies.

## Conflict of Interest Statement

The authors reported no conflicts of interest.

The *Journal* policy requires editors and reviewers to disclose conflicts of interest and to decline handling or reviewing manuscripts for which they may have a conflict of interest. The editors and reviewers of this article have no conflicts of interest.

## Supplementary Data

Video 1 Preoperative computed tomography showed acute aortic dissection. Preoperative transesophageal echocardiogram showed left ventricular outflow tract (LVOT) obstruction with systolic anterior motion (SAM) of the mitral valve, and moderate mitral regurgitation (MR). The transaortic Alfieri stitch was applied through the LVOT. Postoperative transesophageal echocardiogram showed corrected SAM and reduced MR. Video available at: https://www.jtcvs.org/article/S2666-2507(26)00115-X/fulltext.
