# Supplementary figures and images for: Transaortic Alfieri stitch for mitral systolic anterior motion in acute type A dissection
Source: JTCVS Tech. 2026 Mar 7;37:102308. doi: 10.1016/j.xjtc.2026.102308 (PMC13261204; doi:10.1016/j.xjtc.2026.102308)

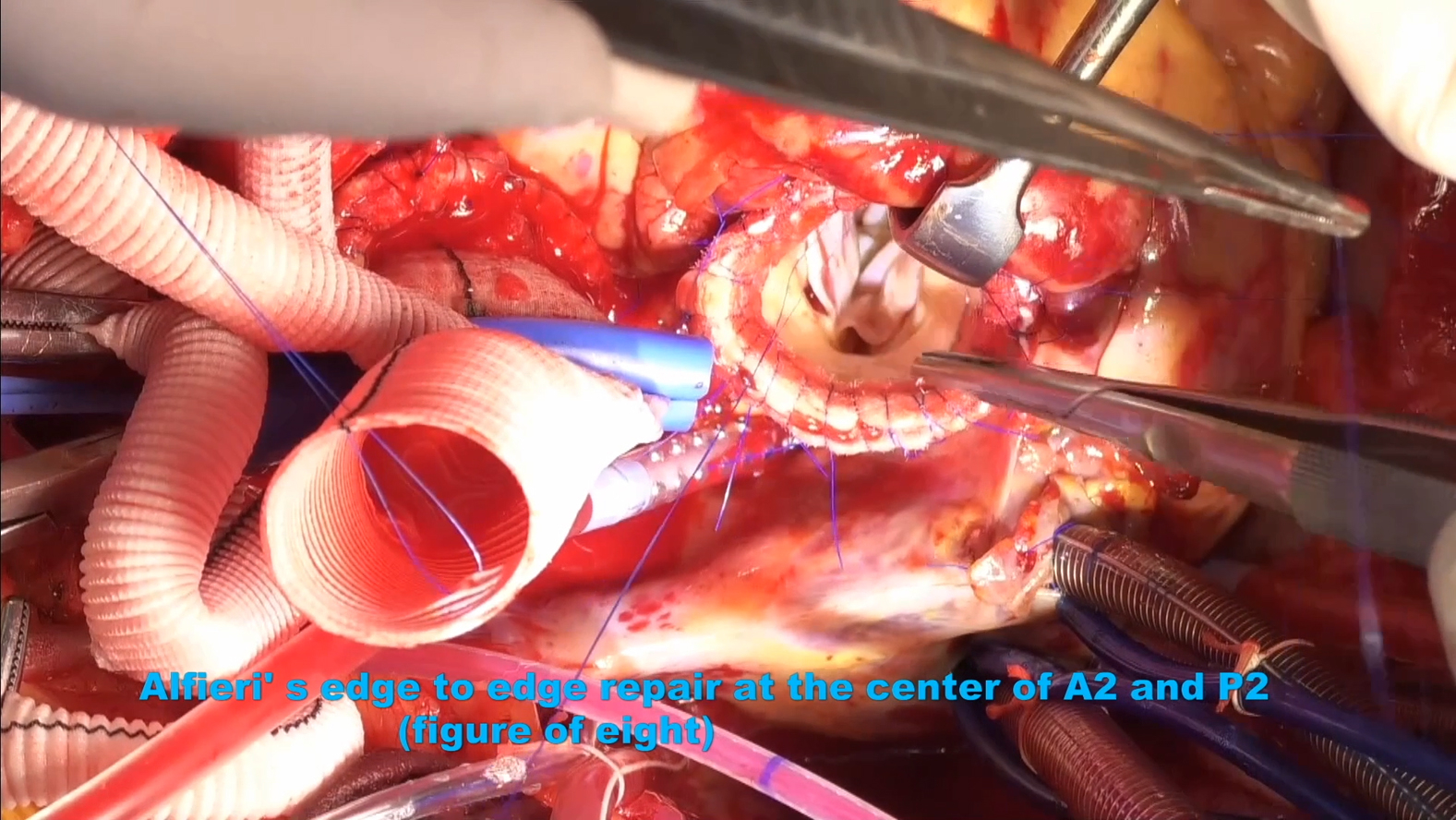

Supplement: Video 1 — Preoperative computed tomography showed acute aortic dissection. Preoperative transesophageal echocardiogram showed left ventricular outflow tract (LVOT) obstruction with systolic anterior motion (SAM) of the mitral valve, and moderate mitral regurgitation (MR). The transaortic Alfieri stitch was applied through the LVOT. Postoperative transesophageal echocardiogram showed corrected SAM and reduced MR. Video available at: https://www.jtcvs.org/article/S2666-2507(26)00115-X/fulltext. [file fx2.jpg]
